# Potential of Compost and Spent Coffee Grounds as Sources of Humic-Like Substances: Extraction Modeling and Optimization by Fractional Factorial Design

**DOI:** 10.1021/acsomega.5c01766

**Published:** 2025-04-11

**Authors:** Dominik Nieweś, Kinga Marecka

**Affiliations:** Department of Engineering and Technology of Chemical Processes, Faculty of Chemistry, Wroclaw University of Science and Technology, Wybrzeże Wyspiańskiego 27, 50-370 Wroclaw, Poland

## Abstract

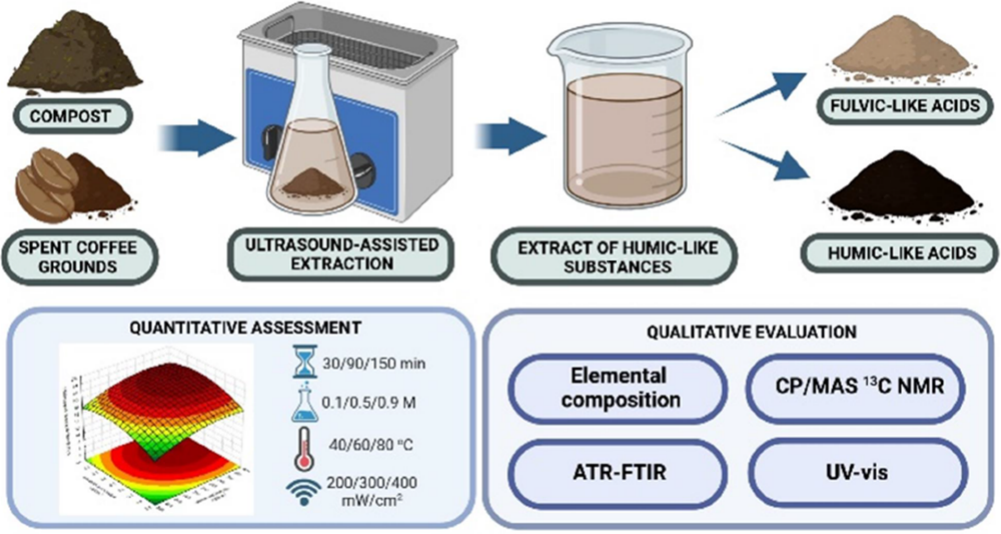

Humic substances (HSs) play a crucial role in soil health and have potential applications beyond agriculture. This study evaluates the renewable raw materials such as compost from urban green waste (GWC) and spent coffee grounds (SCG) as sources of humic-like substances (HLSs), which may be an alternative to traditional resources such as peat, lignite, and leonardite. The quantitative analysis focused on modeling and optimizing the efficiency of ultrasound-assisted extraction of humic-like acids (HLAs) as a function of time, ultrasound intensity, extractant concentration (NaOH), and temperature. Experimental points were determined based on the experimental matrix according to fractional factorial design (FFD), and the obtained polynomial models were evaluated using the Fisher test, lack of fit assessment, and determination coefficients. Experimental verification with optimal values of the evaluated parameters demonstrated the possibility of extracting HLAs from GWC and SCG at levels of 27.08 and 19.11%, respectively. The qualitative analysis included humic-like and fulvic-like acids and their comparison with reference samples. Spectroscopic methods (UV–vis, ATR-FTIR, CP/MAS, and ^13^C NMR) as well as elemental analysis (CHNSO) were used in this part of the study. The results obtained showed the presence of functional groups characteristic of humic substances (polysaccharides, phenols, alcohols, and amides) in the molecular structure of the products. Additionally, they exhibited a higher content of nitrogen and sulfur compared to commercial samples, as well as lower hydrophobicity, which may indicate their biological activity.

## Introduction

Humic substances (HSs) are defined as supramolecular structures that are associated with small molecules, such as proteins, long-chain hydrocarbons, fatty acids, esters, and amino acids, derived from the decomposition of dead biological material.^1,2^ HSs cover aggregates with a broad range of molecular weights, from hundreds to millions of Daltons,^3^ and are divided into three main fractions, differing in solubility at different pH ranges. Fulvic acids (FAs), which are soluble in all pH ranges; humic acids (HAs), which are insoluble at pH < 2; and humins (Hs), which are insoluble under both acidic and alkaline conditions.^4^

Due to their heterogeneous molecular structure, HSs have properties responsible for maintaining proper soil conditions, which determine their main applications in agriculture. Thanks to their buffering properties, an appropriate content of HSs in the soil profile ensures the maintenance of an adequate pH level.^5^ Moreover, HSs may also prevent water evaporation and counteract soil degradation by stabilizing the aggregates of soil particles.^6^ The molecular structure of HSs, which includes numerous functional groups, improves the cation-exchange capacity of the soil, which affects the availability of micronutrients through chelation, as well as the immobilization of heavy metals, thereby reducing their bioavailability to plant root systems.^7,8^ The cation-exchange properties are one of the main reasons for the nonagricultural applications of HSs. Marcinčák et al. described the influence of HSs application as feed additives in the diet of chickens on the quality of the produced meat. The supplementation of HSs with a high proportion of HAs had a positive effect on protein digestion and the optimal utilization of calcium and trace elements, which resulted in increased protein content and lower fat content in the poultry meat produced, as well as improved its oxidative stability.^9^ Moreover, the positive effect of HSs supplementation on the digestibility of proteins and fats in growing pigs was also observed, which may have led to a reduction in ammonia emissions from pig farms.^10^ HSs may also have surfactant properties, allowing them to affect the solubility of organic compounds as well as enhance their bioavailability for microorganisms. This may determine the use of HSs as additives to increase the biodegradation of polycyclic aromatic hydrocarbons.^11,12^ Swaraj Acharya and Bhusan Parida pointed to the possibility of HAs application as a bioorganic catalyst, which, due to their high biodegradability, recyclability, and reusability, may be considered a mild acidic catalyst, which has been tested, among others, in aldol condensation, tetrazole synthesis, hydrogenation, and cross-coupling reactions.^13^

The potential application of humic substances in various sectors is linked to increasing demand and growing market value. In the case of humic acids, the annual growth rate in 2018 was 14%, while for fulvic acids, in 2019, it was 3.5%, with forecasts predicting further dynamic growth in this sector.^14^ Regarding the technology for obtaining humic substances, the main step related to the isolation of HAs and FAs is carried out through alkaline extraction. The traditional procedure, described by Swift and recommended by the International Humic Substances Society, recommends conducting the extraction by intermittent shaking and proposes some additional steps aimed at the preparation of solid-state raw material, as well as the purification of obtained products.^15^ However, this often leads to unsatisfactory yields in obtaining individual fractions in relation to the mass of raw material used, which is related to the low efficiency of mass transfer and the loss of part of the products isolated due to their multistage purification.^16^ Therefore, in recent years, there has been an increasing number of studies on the application of modified extraction methods, such as ultrasound-assisted (UAE) or microwave-assisted extraction (MAE), which significantly improve the efficiency of the process and allow for obtaining larger quantities of humic and fulvic acids in relation to the amount of raw material used.^17−19^

Modifications related to the technology of humic substance extraction increasingly focus not only on intensifying the isolation process but also on searching for raw materials that could serve as alternatives to peat, lignite, or leonardite, which are traditionally considered rich sources of humic substances.^20^ In this context, research is increasingly focused on the extraction of artificial humic acids, which are primarily formed as a result of advanced oxidation of waste biomass in hydrothermal processes.^21,22^ These techniques may, to some extent, serve as an alternative to traditional humification and facilitate the faster transformation of raw materials subjected to the process into products with a molecular structure similar to the humic substances that naturally form in the environment.^23^

An alternative method for isolating humic substances from nonrenewable raw materials could also be the direct extraction of humic-like substances (HLSs), for example, from compost. A valuable source of such substances could also be spent coffee grounds (SCG). The research presented by Cervera-Mata et al. indicates the agricultural potential of SCG as a soil-improving material while also highlighting the need for processing unprocessed SCG to remove harmful compounds. The conducted studies demonstrated the possibility of producing, among other things, biochar and vermicompost, while also pointing out their significant heterogeneity, which often resulted in varying properties in the context of their use as organic soil conditioners.^24^ The positive impact of using SCG as a soil improver is also supported by the results obtained by Comino et al., which suggest an increase in soil organic matter (SOM) content, while also showing that the stability of individual fractions may depend on the type of soil on which SCG was applied.^25^

Application of alkaline extraction without prior treatment with alternative raw materials simplifies the procedure for obtaining HLSs. However, it is often associated with lower efficiency compared to the use of peat or lignite for this purpose.^26^ Therefore, to utilize raw materials with a lower HLSs content, optimizing the process in terms of extraction efficiency is crucial. In the present study, the potential of two alternative and renewable raw materials—compost derived from urban green waste (GWC) and spent coffee grounds (SCG)—was evaluated for the efficient isolation of humic-like acids (HLAs). For this purpose, ultrasound-assisted extraction (UAE) was modeled and optimized to maximize HLA isolation. Additionally, a qualitative analysis of both humic-like and fulvic-like fractions was conducted. The results were compared with the analysis of commercial humic substances, which may help assess the potential of using HLSs obtained from these alternative sources as analogs of classical HSs available on the market.

## Materials and Methods

### Raw Materials and Chemicals

The spent coffee grounds evaluated in this study were derived from Arabica beans and purchased from a local coffee roastery. The SCG was generated after brewing espresso in an automatic coffee machine. To brew a single coffee, 50 mL of water at a temperature of 90 °C was used. For making espresso, approximately 15 g of beans were ground and pressed under a pressure of 15 bar. The obtained SCGs were mixed to create a homogeneous and representative material. The initial moisture content of the raw material was approximately 35%. Before extraction, the SCG was dried at 105 °C for 24 h to prevent microbial spoilage. The raw material thus prepared was stored at room temperature in tightly sealed polypropylene containers until use. The second raw material evaluated was commercially available compost derived from urban green waste (GWC). Due to its high moisture content (approximately 45%), GWC was dried under the same conditions as SCG before the extraction step. It was then ground, sieved through a 1 mm mesh, and stored in a tightly sealed polypropylene container for further investigation.

The HLSs were isolated using NaOH solutions of different concentrations, which were prepared by dissolving solid NaOH. The HCl solution, used for the precipitation of HLAs, was prepared from concentrated acid (38 wt %). The Ba(OH)_2_ and (CH_3_COO)_2_Ca solutions, used for determining total and carboxylic acidity, were also prepared by dissolving the respective compounds in water. All reagents used in this study were of analytical grade and were purchased from Chempur (Piekary Śląskie, Poland). Demineralized water with a conductivity below 1 μS/cm was used to prepare the solutions.

### Extraction and Fractionation Procedure

The basis of the methodology for isolating HLAs in the present study was the procedure proposed by Swift, which is recommended by the International Humic Substances Society (IHSS), as well as the methodology described in the ISO 19822:2018 standard.^15,27^ However, modifications were introduced to enhance the efficiency of the extraction process and minimize the moisture content in the raw materials, thus improving the water balance of the process.

Ten grams of the prepared raw material were placed in a 250 cm^3^ Erlenmeyer flask and mixed with 100 cm^3^ of NaOH solution. The alkaline extraction process was carried out using a thermostated ultrasonic bath. Process parameters, such as the extractant concentration, time, temperature, and ultrasound intensity, were variable and took values defined in the experimental matrix, as described in the “Design of experiment” section (Table 1). The alkaline extract was then separated from the solid residue by centrifugation (3500 rpm, 15 min, room temperature), and the solid particles remaining suspended in the liquid phase were removed by vacuum filtration. The obtained extract was acidified to pH 1 by adding a 6 M HCl solution and then stored for 24 h at 5 °C until the complete precipitation of the HLAs fraction. For quantitative analysis, the gel was separated from the liquid phase by filtration using hard quantitative filter paper. The extraction efficiency for each experimental point was determined as the mass ratio of the obtained HLAs to the raw material used, converted to a dry and ash-free state. For this purpose, the filter papers containing the HLA gel were placed in porcelain crucibles and then dried at 105 °C and incinerated at 650 °C until a constant mass was obtained. The moisture and ash contents for the raw materials used were determined according to the same procedure as for HLAs.

**Table 1:** Factors, Levels, and Values Used in the FFD

factor	name	unit	levels		
			low (−1)	mean (0)	high (1)
*x*_1_	time	min	30	90	150
*x*_2_	ultrasound intensity	mW·cm^–2^	200	300	400
*x*_3_	extractant concentration	mol·dm^–3^	0.1	0.5	0.9
*x*_4_	temperature	°C	40	60	80

Qualitative analysis of HLAs was performed for the sample obtained under optimal extraction conditions, characteristic of maximization of the amount of their isolation efficiency. In this part of the study, FLAs were also evaluated. The fulvic-like acids were isolated from the solution after the precipitation and separation of the humic-like fraction. For this purpose, the liquid phase was passed through the column filled with Supelite DAX-8 resin, with the volumetric flow equaling 4-5 cm^3^·min^–1^. The subsequent steps included washing the column with demineralized water, followed by the desorption of the FLA fraction through backwashing the column with 0.1 M NaOH. The obtained eluate was subjected to a protonation process by passing it through an acidic ion-exchange resin. The resulting liquid phase was concentrated to a volume of approximately 50 cm^3^ using a rotary vacuum evaporator. The final step involved complete evaporation of water at 105 °C until a constant FLA fraction was obtained.

### Design of Experiment

In the presented work, the influence of four process parameters, namely, extraction time (x_1_), ultrasound intensity (x_2_), extractant concentration (x_3_), and temperature (x_4_), on the extraction efficiency of HLAs from GWC and SCG was examined. For this purpose, a fractional factorial design (FFD) was applied, and each process parameter was coded at three levels (Table 1). The statistical analysis, based on analysis of variance (ANOVA), was carried out using Statistica 13.3 software (TIBCO Software Inc., Palo Alto, CA). The influence of the examined parameters on HAs isolation efficiency from the assessed raw materials was determined through the effect estimates. The adequacy of the polynomial models describing the response, as a function of the tested independent variables, was assessed based on the coefficients of determination, the lack of fit test, and Fisher’s test.

### Qualitative Analysis

The molecular structure characterization, identification of functional groups, and carbon-type distribution of the humic samples were assessed using ultraviolet–visible spectroscopy (UV–vis), attenuated total reflection-Fourier transform infrared spectroscopy (ATR-FTIR), and solid-state cross-polarization magic angle spinning carbon-13 nuclear magnetic resonance (CP/MAS ^13^C NMR). Moreover, the qualitative analysis of humic-like and fulvic-like samples included elemental analysis as well as potentiometric determination of total acidity and carboxyl group content.

### UV–Vis

UV–vis spectra were recorded by using a JASCO V-670 spectrophotometer with a scan range of 200–800 nm and a scan resolution of 0.2 nm. For this purpose, 0.1 mg of the solid sample was dissolved in 100 cm^3^ of phosphate buffer by shaking at room temperature for 24 h. The supernatant was then filtered using syringe filters and measured. The baseline was corrected by using deionized water.

### ATR-FTIR

The ATR-FTIR spectra were collected by using a Bruker Vertex 70 spectrometer at room temperature. For this purpose, the humic sample was mixed with KBr in a mass ratio of 1:50 and prepared as pellets. The spectra were acquired in the wavenumber range from 4000 to 400 cm^–1^, with a resolution of 4 cm^–1^.

### CP/MAS ^13^C NMR

The NMR measurements were performed by using a Bruker Avance III spectrometer operating at a frequency of 300 MHz and equipped with a 4 mm wide-bore MAS probe. The spectrometer was set to the ^13^C resonance frequency of 75.45 MHz, with the sample rotating at 10 kHz. A total of 4096 data points were collected during the measurement. The acquisition time and recycle delay were set to 50 ms and 4 s, respectively. The CP/MAS ^13^C NMR spectra were recorded over a chemical shift range of 0–220 ppm and quantified by integrating the regions as follows: alkyl-C (0–50 ppm), O-alkyl-C (50–110 ppm), aromatic-C (110–145 ppm), phenol-C (145–163 ppm), carboxyl-C (163–190 ppm), and carbonyl-C (190–220 ppm). The distribution of carbon in different structural groups was quantified by calculating the ratio of each region to the entire shift range, following the methodology outlined by Xu et al.^28^ Additionally, following Weber et al., the hydrophobicity (HB) of the tested samples was determined as the ratio of the sum of alkyl-C and aromatic-C regions (0–50 and 110–145 ppm) to the sum of all other carbon distributions (50–110 ppm; 145–163 ppm; 163–190 ppm, and 190–220 ppm).^29^

### CHNSO Analysis

The bulk content of elements was identified using the Vario EL Cube analyzer. For each sample, measurements were performed in triplicate to obtain the most precise results, and the data are presented as the mean with the standard error. For calibration, acetanilide and sulphanilamide were used as the standards. The contents of carbon (C), hydrogen (H), nitrogen (N), and sulfur (S) are presented as mass percentages of the dry and ash-free state, while the oxygen (O) content was calculated as the difference. For this purpose, the samples were dried at 105 °C before analysis, and the ash content was determined by calcining them at 650 °C to a constant weight.

### Potentiometric Titration

The total acidity and carboxylic group content of the samples were determined using the method described by Schnitzer and Gupta.^30^ In the case of determining total acidity, the method is based on the reaction with Ba(OH)_2_, the excess of which is titrated using a standardized 0.1 M HCl solution, with the end point assumed to be at a pH of 8.4. The determination of carboxylic acidity is based on reaction with (CH_3_COO)_2_Ca and the substitution of functional groups in humic substances with Ca^2+^ ions, leading to the liberation of acetic acid, which is then titrated with a standardized 0.1 M NaOH solution. In this case, the endpoint of the titration is set at a pH of 9.8. For the potentiometric titration of the samples, a HANNA HI 932 automatic titrator equipped with a pH electrode was used. The phenolic–OH group content was calculated as the difference between the total acidity and the COOH content.

## Results and Discussion

### Statistical Analysis

The experimental matrix designed in this study is presented in Table 2. According to the FFD for the defined experimental space, 27 runs were necessary. However, to estimate the pure error for the lack of fit test and assess the stability of the conducted experiments, the matrix was expanded to include a central point, which was performed three times. As a result, a total of 30 experiments were conducted in one block, and the sequence of runs was randomized to minimize the influence of uncontrolled factors on the response. The extraction efficiency for the experimental points included in the designed matrix ranged from 4.65 to 24.25% for HLA isolation from GWC, and from 2.40 to 17.26% when SCG was tested as the raw material.

**Table 2:** Efficiency of HLAs Isolation from GWC and SCG for the Experimental Runs According to FFD

run	variables (coded)				responses	
	time (min)	ultrasound intensity (mW·cm^–2^)	extractant concentration (mol·dm^–3^)	temperature (°C)	efficiency of HLAs extraction from GWC (%)	efficiency of HLAs extraction from SCG (%)
1	–1	–1	–1	–1	4.65	2.40
2	–1	–1	0	1	16.32	11.03
3	–1	–1	1	0	14.43	10.68
4	–1	0	–1	1	13.12	8.63
5	–1	0	0	0	18.05	12.85
6	–1	0	1	–1	11.71	10.81
7	–1	1	–1	0	14.46	9.69
8	–1	1	0	–1	16.02	12.26
9	–1	1	1	1	21.15	15.22
10	0	–1	–1	1	16.44	11.95
11	0	–1	0	0	15.36	14.63
12	0	–1	1	–1	12.29	11.48
13	0	0	–1	0	19.12	13.49
14	0	0	0	–1	19.38	12.46
15	0	0	1	1	20.35	14.71
16	0	1	–1	–1	13.24	8.92
17	0	1	0	1	24.25	15.07
18	0	1	1	0	20.61	13.94
19	1	–1	–1	0	17.02	10.35
20	1	–1	0	–1	16.49	11.69
21	1	–1	1	1	20.74	16.09
22	1	0	–1	–1	17.21	12.45
23	1	0	0	1	23.16	17.07
24	1	0	1	0	23.38	14.21
25	1	1	–1	1	21.15	11.97
26	1	1	0	0	20.85	15.28
27	1	1	1	–1	18.10	12.14
28 (C)	0	0	0	0	24.04	16.54
29 (C)	0	0	0	0	22.86	17.26
30 (C)	0	0	0	0	23.43	16.05

### Effect Estimates and Response Surfaces

The influence of changes in the values of the studied process variables on the response was determined by the effect estimates. Effects were considered significant if the p-value was lower than 0.05.^31^ For both evaluated raw materials, 9 significant effects were identified. For the extraction process using GWC (Table 3), all linear and quadratic effects were significant as well as the interaction effect between time and ultrasound intensity (*x*_1_·*x*_2_). When SCG was used as the raw material (Table 4), significant effects for the process included all linear effects, quadratic effects of time (*x*_1_^2^), ultrasound intensity (*x*_2_^2^), and NaOH concentration (*x*_3_^2^), as well as interaction effects between time and ultrasound intensity (*x*_1_·*x*_2_), and time and extractant concentration (*x*_1_·*x*_3_). All significant linear and quadratic effects in this study had positive values, suggesting their positive impact on the HLAs extraction yield. Considering their values, for the extraction process using GWC, the dominant linear effects were the influence of time, temperature, and the intensity of the generated ultrasound. Data for the HLAs isolation process from SCG also indicate higher values for linear effects, with the dominance of the effect of time, extractant concentration, and temperature. However, in that case, the difference between these values and the quadratic effects is smaller than in the case of extraction from compost.

**Table 3:** Effect Estimates of the Tested Factors on the Efficiency of HLAs isolation from GWC

parameter	effect	standard error	confidence interval		*p*-value	remarks
			–95%	+95%		
*x*_1_	**5.35**	**0.28**	**4.16**	**6.55**	**0.003**	**significant**
*x*_1_^2^	**1.16**	**0.23**	**0.18**	**2.14**	**0.036**	**significant**
*x*_2_	**4.01**	**0.28**	**2.81**	**5.21**	**0.005**	**significant**
*x*_2_^2^	**1.90**	**0.23**	**0.92**	**2.88**	**0.014**	**significant**
*x*_3_	**2.93**	**0.28**	**1.73**	**4.12**	**0.009**	**significant**
*x*_3_^2^	**2.63**	**0.23**	**1.65**	**3.61**	**0.007**	**significant**
*x*_4_	**5.28**	**0.28**	**4.09**	**6.48**	**0.003**	**significant**
*x*_4_^2^	**1.53**	**0.35**	**0.55**	**2.51**	**0.021**	**significant**
*x*_1_·*x*_2_	**–1.64**	**0.35**	**–3.15**	**–0.13**	**0.043**	**significant**
*x*_1_·*x*_3_	–1.50	0.35	–3.01	0.01	0.051	not significant
*x*_1_·*x*_4_	–0.81	0.35	–2.32	0.71	0.149	not significant
*x*_2_·*x*_3_	0.07	0.35	–1.44	1.59	0.852	not significant
*x*_2_·*x*_4_	–0.52	0.35	–2.04	0.99	0.276	not significant
*x*_3_·*x*_4_	0.34	0.35	–1.17	1.86	0.431	not significant

**Table 4:** Effect Estimates of the Tested Factors on the Efficiency of HLAs Isolation from SCG

parameter	effect	standard error	confidence interval		*p*-value	remarks
			–95%	+95%		
*x*_1_	**3.08**	**0.29**	**1.84**	**4.31**	**0.001**	**significant**
*x*_1_^2^	**1.22**	**0.23**	**0.21**	**2.22**	**0.009**	**significant**
*x*_2_	**1.58**	**0.29**	**0.34**	**2.81**	**0.035**	**significant**
*x*_2_^2^	**1.22**	**0.23**	**0.21**	**2.23**	**0.032**	**significant**
*x*_3_	**3.27**	**0.29**	**2.04**	**4.50**	**0.035**	**significant**
*x*_3_^2^	**2.16**	**0.23**	**1.16**	**3.17**	**0.008**	**significant**
*x*_4_	**3.01**	**0.29**	**1.78**	**4.24**	**0.012**	**significant**
*x*_4_^2^	0.96	0.23	–0.04	1.97	0.055	not significant
*x*_1_·*x*_2_	**–1.97**	**0.36**	**–3.53**	**–3.53**	**0.032**	**significant**
*x*_1_·*x*_3_	**–1.68**	**0.36**	**–3.24**	**–3.24**	**0.044**	**significant**
*x*_1_·*x*_4_	–0.22	0.36	–1.78	–1.79	0.598	not significant
*x*_2_·*x*_3_	–0.52	0.36	–2.09	–2.09	0.283	not significant
*x*_2_·*x*_4_	–1.18	0.36	–2.74	–2.74	0.083	not significant
*x*_3_·*x*_4_	–0.02	0.36	–1.58	–1.59	0.952	not significant

**Figure 1:**
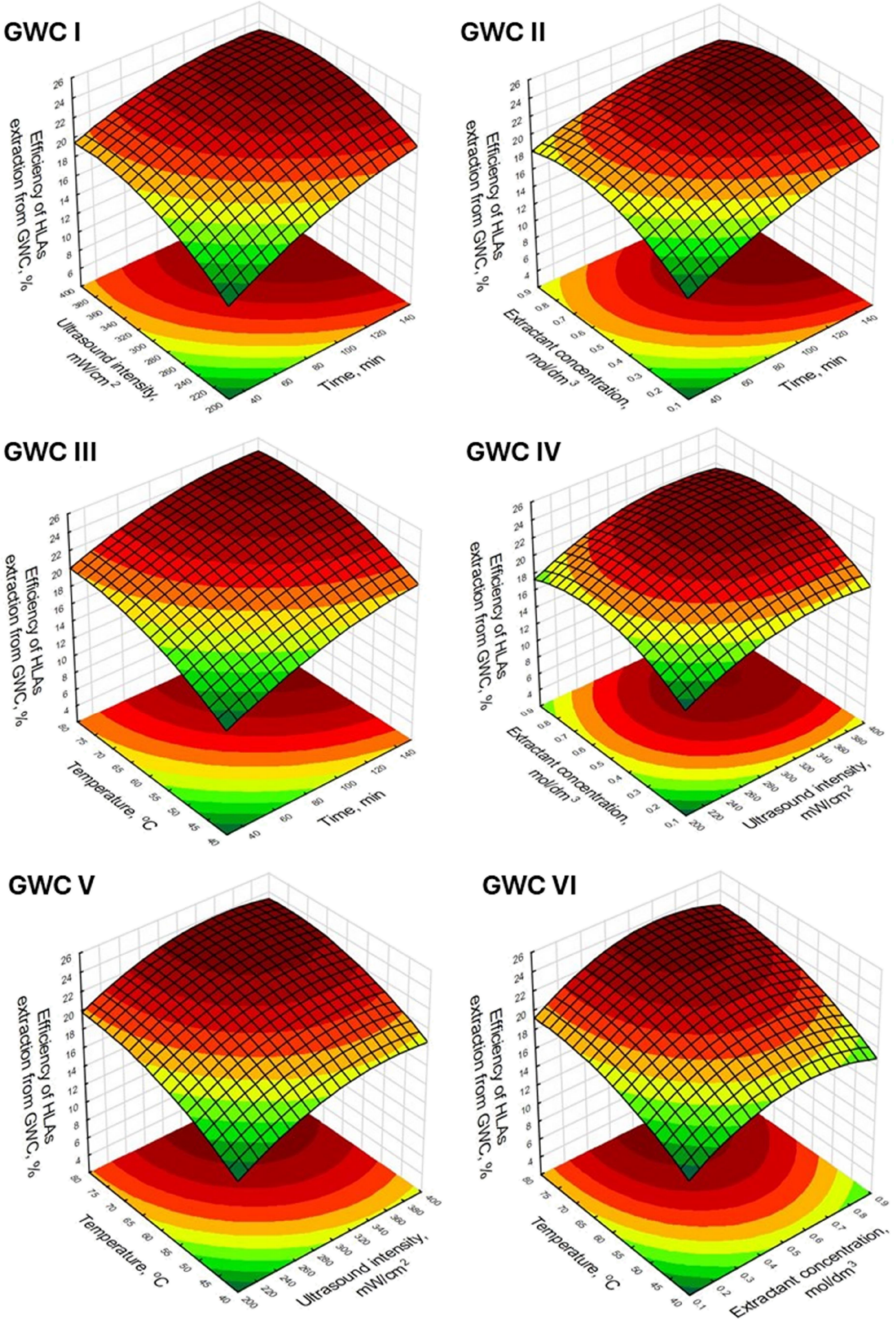
Response surface plots of the influence of tested process parameters on the extraction efficiency of HLAs from GWC.

**Figure 2:**
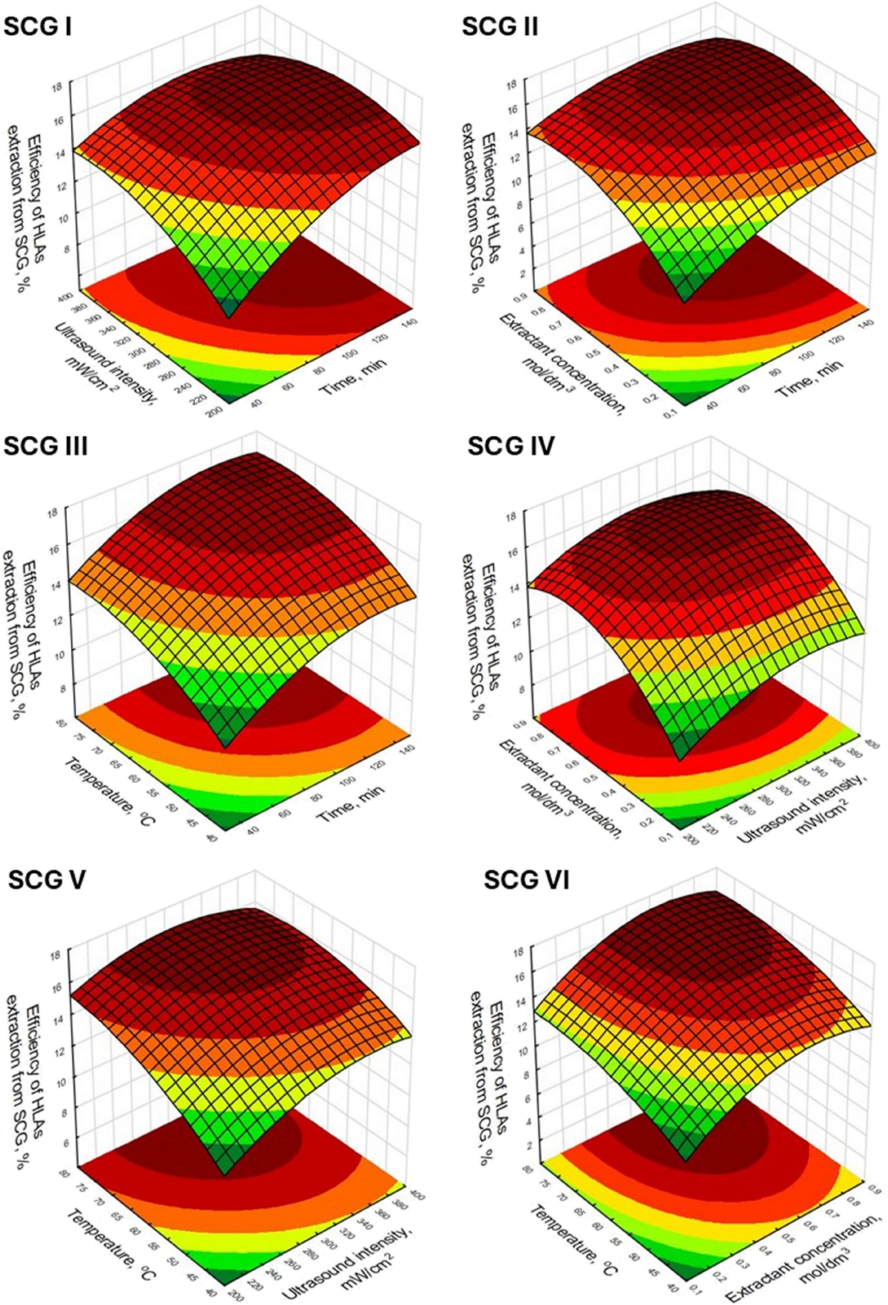
Response surface plots of the influence of tested process parameters on the extraction efficiency of HLAs from SCG.

The graphical representation of the relationship between the values of the studied process factors and the HLA isolation yield is presented using response surface plots. On the single graph, the change in extraction efficiency as a function of two of the four evaluated parameters is presented, with the values of the other two parameters, not shown in the plot, were defined as constant and coded as 0. Overall, the shapes of the graphs for the process using GWC (Figure 1) and SCG (Figure 2) as the source of HLAs were similar. This is because similar effects were considered significant for both of the evaluated raw materials. However, in the case of the HLAs extraction process from GWC, a more dynamic increase in efficiency is observed as the process parameter values increase. This corresponds to higher values of statistically significant effects outlined in Table 3, than in the case of extraction from SCG (Table 4). Moreover, under identical extraction conditions, a higher degree of HLA isolation was achieved when GWC was used as the raw material, as shown by the results in Table 2, where this pattern was observed at every experimental point in the matrix.

The relationship between the evaluated independent variables and the extraction efficiency of HLAs from GWC (*Y*_GWC_) and SCG (*Y*_SCG_) is described by eqs 1 and 2, respectively. They refer to the coded values of the process parameters and include only those elements whose effects on the extraction efficiency were statistically significant.
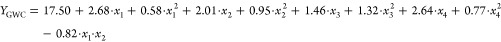
1
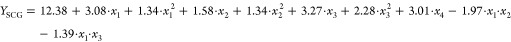
2

The generated models were used for the determination of optimal process conditions for the maximization of the extraction efficiency of HLAs from raw materials evaluated. The results of optimization are presented in Table 5. All of the determined values fell within the boundaries defining the experimental space, which may indicate that it was properly designed. A simulation employing polynomial equations and optimal parameters demonstrated that an HLAs extraction efficiency of 25.87% can be achieved when using GWC as the raw material and 18.74% when the extraction is carried out using SCG. Experimental verification indicated slightly higher values, amounting to 27.08 and 19.11% for the processes using GWC and SCG as raw materials, respectively.

**Table 5:** Optimal Values of the Process Parameters for the Maximalization of HLAs Yield Isolated from GWC and SCG

raw material type	process parameters			
	time, min	ultrasound intensity, mW·cm^–2^	extractant concentration, mol·dm^–3^	temperature, °C
compost from green wastes	142	330	0.57	75
spent coffee grounds	122	288	0.61	76

### Analysis of Variance and Models Fitting

**Figure 3:**
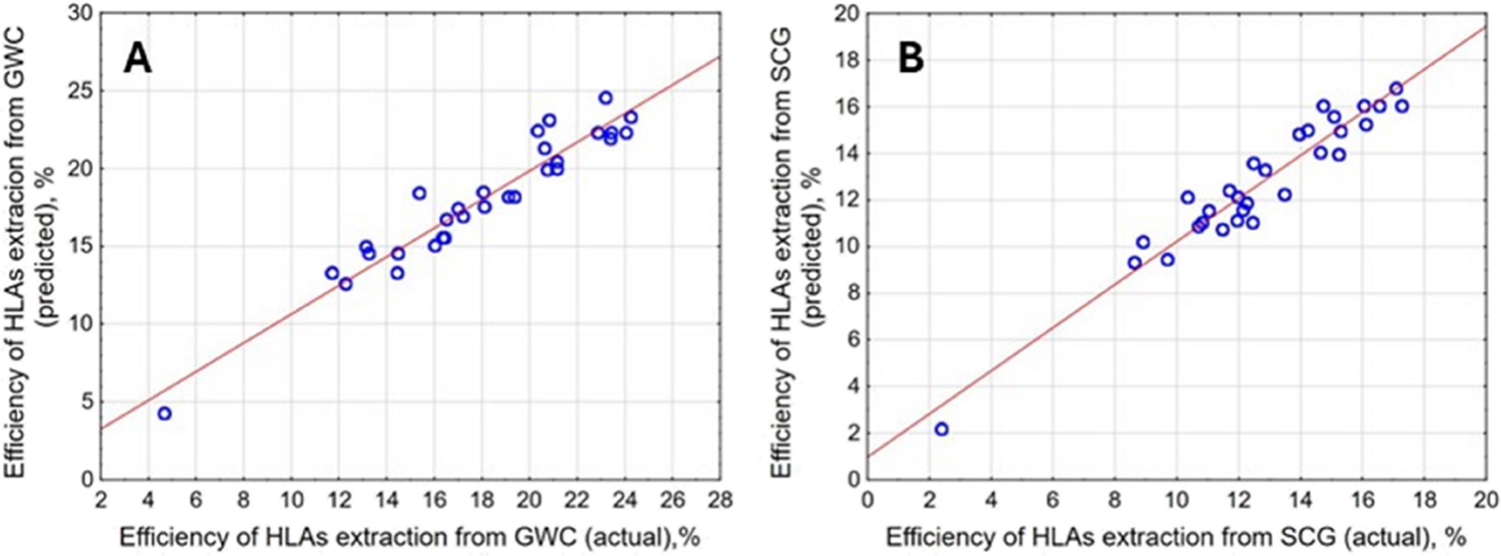
Actual vs predicted efficiency of HLAs extraction from GWC (A) and SCG (B).

A good model fit is indicated by the relationships between the experimental and predicted data (Figure 3), which show only minor discrepancies. However, detailed evaluation of the reduced models’ suitability for predicting outcomes within the considered experimental space was conducted based on an analysis of variance (ANOVA), the results of which are presented in Table 6. The calculated Fisher test coefficients (*F*-value) are significantly higher than the pure error in both cases, while the p-values for the lack of fit are greater than 0.05. This indicates that the presented polynomial models are well-fitted to the data within the studied experimental space.^32^ Moreover, the calculated *F*-values (19.16 and 15.31) are significantly higher than the standard *F*-Snedecor distribution value, which for both cases equals 2.39. The coefficients of determination (*R*^2^) were 90.50 and 92.37% for the models describing the extraction efficiency of HLAs from GWC and SCG, respectively. These results indicate that for both investigated processes, the percentage of variances unexplained by the polynomial eqs (eqs 2 and 3) is below 10%. An *R*^2^ value above 90% is commonly considered an indication of the suitability of polynomial models for predicting experimental outcomes.^33,34^

**Table 6:** Results of Analysis of Variance (ANOVA) for Extraction Efficiency

source	sum of square (SS)	degree of freedom (df)	mean square (MS)	*F*-value	*p*-value
extraction of HLAs from GWC					
model	470.73	9	52.30	19.16	0
residual	54.68	20	2.73		
lack of fit	53.98	18	3.00	8.61	0.11
pure error	0.70	2	0.35		
extraction of HLAs from SCG					
model	220.50	9	24.50	15.31	0
residual	31.90	20	1.60		
lack of fit	31.16	18	1.73	4.67	0.19
pure error	0.74	2	0.37		

### Humic Fraction Analysis

In the qualitative assessment section, a total of 6 samples were analyzed. Four of them were fulvic-like (FLAs) and humic-like (HLAs) fractions, which were isolated under optimal extraction conditions, ensuring the maximum degree of humic-like acid isolation within the experimental space studied (Table 5). The second part of the sample designations refers to the raw material from which the individual fractions were derived: green waste compost (GWC) or spent coffee grounds (SCG). Complementing the qualitative assessment, analyses were also performed for commercial humic acids (HAs_SA) and sodium humates (NaHAs_SA), which were purchased from Sigma-Aldrich.

### Elemental Analysis and Acidity

Table 7 presents the results of the analysis of carbon, hydrogen, nitrogen, sulfur, and oxygen contents in the raw materials from which HLSs were extracted in this study. Generally, the properties of compost related to its elemental composition, as well as the composting process itself, are largely dependent on the type of raw material subjected to composting. Therefore, the properties of the obtained products may vary significantly.^35^ In the case of SCG composition, the results obtained in this study were similar to those reported by other authors.^36,37^ GWC was characterized by a higher mass fraction of carbon and nitrogen compared to SCG, while the hydrogen content was similar. Additionally, SCG exhibited a higher oxygen content compared to that of the second raw material tested.

**Table 7:** Results of CHNSO Analysis for the Raw Materials Evaluated in This Study

raw material type	elemental composition, %				
	C	H	N	S	O
GWC	59.28	6.86	5.54	2.08	26.24
	±5.46	±1.52	±1.01	±0.24	±2.44
SCG	54.71	7.13	2.98	1.25	33.93
	±4.13	±1.04	±0.86	±0.38	±3.86

The elemental composition and acidity of the humic and fulvic samples are summarized in Table 8. In general, the isolated samples in this study, compared to the commercial HAs, exhibited a higher proportion of heteroatoms, particularly nitrogen (N). This can be attributed to the increased presence of the protein structures. Moreover, the nitrogen content was higher for the HLAs, which may indicate the coprecipitation of proteins with the humic-like fraction during its isolation from the alkaline solution.^38^ When analyzing the results for individual fractions, FLAs contained a lower carbon (C) content, while the percentages of hydrogen (H) and oxygen (O) were higher. This trend was consistent regardless of the type of raw material.

**Table 8:** Elemental Composition and Acidity of Samples Evaluated

sample	elemental composition, %					acidity, mmol·g^–1^		
	C	H	N	S	O	total	carboxylic	phenolic
FLAs_GWC	55.12	5.71	3.52	1.60	34.05	7.68	3.75	3.93
	±2.12	±1.08	±0.82	±0.32	±2.21	±1.11	±0.85	±0.71
FLAs_SCG	54.93	6.08	4.83	1.10	33.06	8.28	4.12	4.16
	±1.21	±1.79	±1.04	±0.43	±1.93	±2.12	±0.88	±0.68
HLAs_GWC	60.37	5.14	5.44	1.75	27.30	9.00	4.71	4.29
	±2.42	±0.89	±1.26	±0.68	±3.07	±0.98	±0.43	±0.38
HLAs_SCG	61.45	4.97	6.17	1.22	26.19	9.45	5.43	4.02
	±3.11	±0.44	±1.02	±0.18	±3.03	±1.52	±0.54	±1.06
HAs_SA	64.70	4.08	1.16	0.58	29.48	7.11	3.85	3.26
	±3.27	±0.24	±0.53	±0.23	±2.54	±1.13	±0.87	±0.69
NaHAs_SA	66.45	3.88	1.29	0.55	27.83	3.26	1.04	2.22
	±2.45	±1.30	±0.43	±0.15	±1.62	±0.68	±0.32	±0.49

Regarding the acidity of the samples, the HLAs and FLAs obtained in this study exhibited a higher total acidity compared to the reference samples (HAs_SA and NaHAs_SA). When the individual fractions were compared, humic-like acids were characterized by higher carboxylic acidity. The phenolic acidity in all extracted samples was higher than that in the humic acids and sodium humates from Sigma-Aldrich; however, no significant differences in this parameter were observed between the extracted samples.

### ATR-FTIR

**Figure 4:**
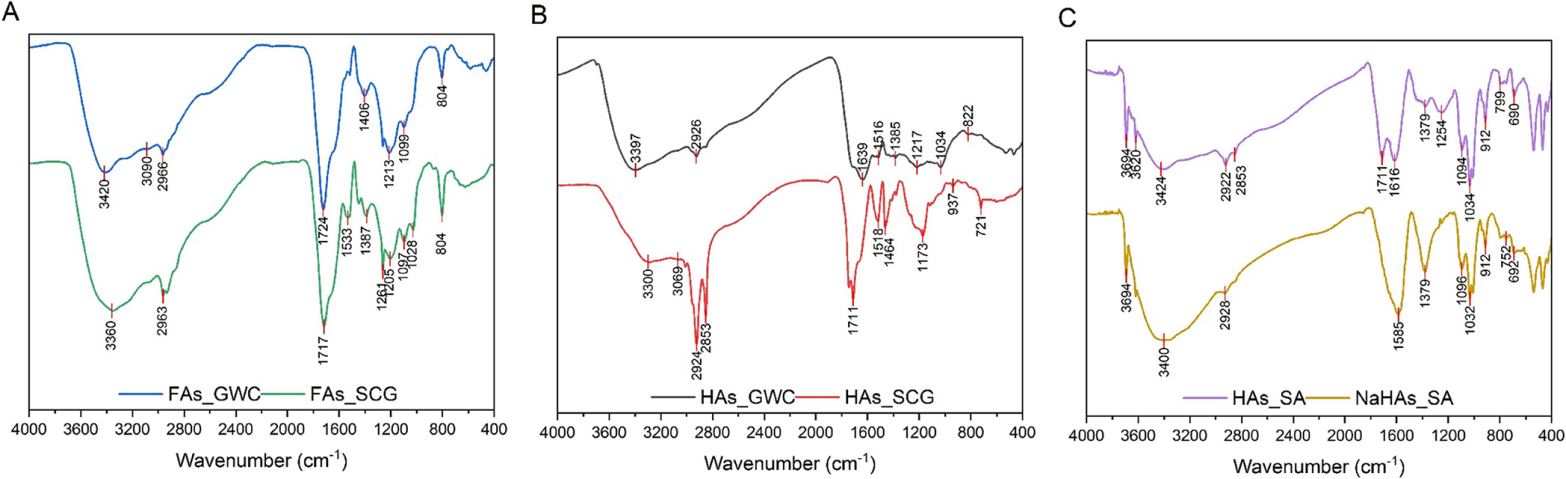
ATR-FTIR spectra of fulvic-like (A) and humic-like (B) acids isolated from GWC and SCG, as well as reference samples (C).

The ATR-FTIR spectra of the tested samples are shown in Figure 4. They confirmed the presence of structures that are commonly observed in humic substances. The broad band, peaking at 3400 cm^–1^, corresponds to the −OH vibrations of phenols, alcohols, carboxylic acids, as well as hygroscopic water.^39,40^ Small peaks at 2920 and 2850 cm^–1^ are attributed to the stretching of aliphatic C–H bonds in methylene (CH_2_) and methyl (CH_3_) groups.^41^ The bands at 1720 and 1620 cm^–1^ correspond to the C=O stretching of carboxyl and aromatic substituents, respectively.^42,43^ The signal identified at about 1380 cm^–1^ indicates the presence of dissociated carboxylic groups (COO^–^), as well as bending deformation of O–H.^44,45^ The peak at 1220 cm^–1^ is associated with the C–O vibration of aryl ethers.^46^ The signals in the area of 1200–1000 cm^–1^ result from C–O to C–O–C vibrations in polysaccharides, alcohols, ethers, and phenols.^47^ The bands below 1000 cm^–1^ are mainly characteristic of vibrations in aromatic structures, such as the C–H deformation out of plane in the aromatic ring (900–650 cm^–1^ region).^48^

### UV–Vis

**Figure 5:**
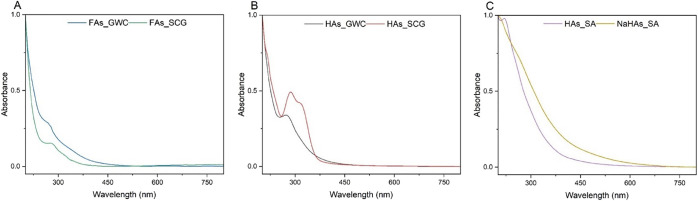
UV–vis absorption spectra of fulvic-like (A) and humic-like (B) acids isolated from GWC and SCG, and reference samples (C).

Absorbance in the UV–vis range during the analysis of humic substances is dependent on the relative content of aromatic structures, chromophores, and auxochromes. For this reason, UV–vis analysis can provide insights into the structural differences between the analyzed humic samples.^49^ The normalized spectra presented in Figure 5 indicate the decrease of absorption by humic and humic-like samples evaluated with the wavelength increase within the tested range. The results for FLAs (Figure 5A) and HLAs (Figure 5B) demonstrated the intensities in the 250–300 nm range, indicating the absorption of light by the double bonds within the aromatic structures.^50^ The peak at about 300 nm, evident for both HLAs and FLAs, but especially intensive for the HLAs_GWC sample, is characteristic of the presence of phenolic compounds.^51^ Moreover, spectra of humic-like acids isolated from compost also exhibited the absorption intensity at 340 nm, which could indicate the *n* → π* and π → π* transitions in H_2_C=CH–CH=O structures.^52^

Based on the UV–vis measurements, four spectral ratios were determined (Table 9), with the indices specifying the wavelengths at which absorption was measured. The coefficients identified in this study allow conclusions about the molecular size and condensation (*E*_250_/*E*_365_), the proportion of the phenolic/quinoid core (*E*_270_/*E*_400_), and the relative amount of lignin (*E*_280_/*E*_472_).^51,53^ The results highlight the differences in the molecular structure of the samples, due to both the raw material used and the type of fraction assessed. The fractions extracted from SCG exhibited lower values of spectral ratios *E*_250_/*E*_365_, *E*_270_/*E*_400_, and *E*_280_/*E*_472_ compared to samples obtained from GWC, which may indicate their lower molecular weight, a reduced degree of structural condensation, and a lower lignin content.

**Table 9:** Absorption Coefficients of Samples Evaluated

sample	*E*_250_/*E*_365_	*E*_270_/*E*_400_	*E*_280_/*E*_472_
FLAs_GWC	3.92	3.68	3.88
FLAs_SCG	3.56	3.38	3.12
HLAs_GWC	4.94	6.17	9.38
HLAs_SCG	4.56	5.85	7.46
HAs_SA	8.30	3.74	4.68
NaHAs_SA	8.08	3.42	5.56

### CP/MAS ^13^C NMR

**Figure 6:**
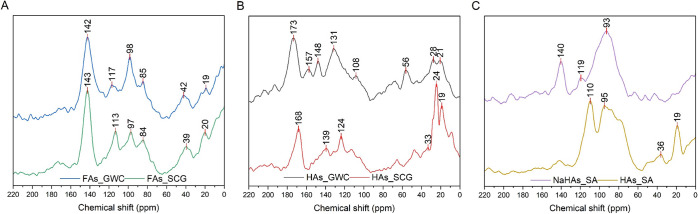
CP/MAS ^13^C NMR spectra of fulvic-like (A) and humic-like (B) acids extracted from GWC and SCG, along with reference samples (C).

The CP/MAS ^13^C NMR spectra of the tested samples (Figure 6) revealed the presence of signals characteristic of the functionalities that are part of the molecular structures of humic substances. Peaks at the resonance range 20–40 ppm, which were especially intense for the HLAs extracted from spent coffee grounds, were attributed to the methine, methyl, and methylene groups.^54^ The signals in the 65–85 ppm area indicated the presence of −CH(OH)– and −CH_2_–O–C– groups.^55^ The shoulders centered around the 100 ppm, derived for O-alkyl anomeric carbon.^56^ The resonances peaking in the 110–145 ppm region may be assigned to the unsaturated carbon as well as aromatic-C substituted with a carboxyl group, carboxymethyl group, and aromatic-C in the meta position relative to the O and N substituents.^57,58^ The resonance, peaking at 155 ppm, indicated the presence of carbon associated with phenolic OH groups.^59^ Finally, the signal at about 170 ppm may be explained by the presence of carboxyl functionalities, with a possible contribution from C in esters and amides.^60,61^

The relative distribution of carbon (Table 10) showed some differences for the evaluated samples, depending on both the type of fraction and the raw material from which HLSs were extracted. First, FLSs were characterized by a higher proportion of O-alkyl structures, compared to HLSs and reference samples, while humic-like fractions were described by the higher distribution of phenolic structures. Moreover, HLAs contained a higher proportion of carboxyl and carbonyl structures, compared to FLAs that were obtained in this study. Taking into account the type of raw material that was used for the isolation of HLSs, the fractions extracted from compost contained a higher percentage of aromatic-C than samples derived from spent coffee grounds, which is especially evident for a humic-like fraction. Similar results in the changes of molecular structure of humic fraction during composting were observed by de Souza et al., who pointed out an increase in the share of aromatic-C accompanied by a simultaneous decrease in the share of aliphatic structures as the composting process progressed.^62^ Simultaneously, when these results are compared with reference samples of humic acids and sodium humates, a greater proportion of aliphatic carbon (0–110 ppm) and carbon bonded with heteroatoms is observed in both HLSs isolated from compost and SCG.

**Table 10:** Percentage of Carbon in the Main Structural Fragments Based on the CP/MAS ^13^C NMR Data and Calculated Hydrophobicity (HB)

sample	distribution of various carbon types, %						HB
	alkyl	O-alkyl	aromatic	phenol	carboxyl	carbonyl	
	0–50	50–110	110–145	145–163	163–190	190–220	
	ppm	ppm	ppm	ppm	ppm	ppm	
FLAs_GWC	33.9	24.8	27.2	5.8	6.1	2.2	1.57
FLAs_SCG	31.5	28.9	24.1	6.2	6.9	2.4	1.25
HLAs_GWC	36.8	15.1	28.5	7.5	9.2	2.9	1.88
HLAs_SCG	38.9	14.1	23.8	9.0	10.3	3.9	1.68
HAs_SA	34.2	14.8	32.4	5.7	8.8	4.1	1.99
NaHAs_SA	31.8	12.9	35.7	4.8	7.9	6.9	2.08

The observed differences influenced the hydrophobicity index (HB), which, according to de Aquino et al., can be used to assess the biochemical stability of humic and humic-like substances.^56^ Aguiar et al. pointed out the relationship between the molecular structure of humic substances and their biological activity, emphasizing the role of hydrophilic carbon in the process of lateral root emergence, while a high hydrophobicity index is negatively correlated with this process.^63^ In this study, all of the extracted samples exhibited lower HB values compared to the commercial samples (HAs_SA and NaHAs_SA). At the same time, differences were noted based on the type of fraction and the raw material from which it was obtained. Generally, the humic-like fraction exhibited a higher HB value than the fulvic-like fraction. Considering the type of raw material, products with higher hydrophobicity indices were obtained from the compost.

## Conclusions

In this study, the potential of using compost derived from urban green waste (GWC) and spent coffee grounds (SCG) as a renewable raw material base for the production of humic-like substances was evaluated. The quantitative research focused on the optimization of ultrasound-assisted extraction of humic acids (HAs) in relation to time (*x*_1_), ultrasound intensity (*x*_2_), extractant concentration (*x*_3_), and temperature (*x*_4_). The analysis of experimental points, determined according to Fractional Factorial Design, showed that the process efficiency was mainly determined by the linear and quadratic effects of the assessed independent variables with positive values for both cases. It was also demonstrated that for the extraction of HLAs from green waste compost (GWC), the interaction between time and ultrasound intensity was significant, while for the process using SCG as the raw material, the interaction between time and extractant concentration was additionally important. For both raw materials, the interaction effects were negative. The generated polynomial models showed a good fit, as confirmed by the Fisher test results and the lack of fit analysis. In both optimization equations, the determination coefficients exceeded 90%, and the optimal values of the evaluated parameters for maximizing the HAs extraction efficiency were within the designed experimental space. Experimental verification of the processes conducted under optimal process parameters showed a higher extraction efficiency of HLAs from GWC, which amounted to 27.08%. For extraction from SCG, it was 19.11%.

The qualitative analysis of humic-like and fulvic-like acids showed the similarities to the results for the commercially available humic reference samples, indicating the presence of polysaccharides, phenols, alcohols, and amides in their molecular structure. However, some differences were also determined. The isolated samples exhibited a higher content of heteroatoms (nitrogen and sulfur), as well as generally higher proportions of aliphatic and phenolic structures. The spectroscopic coefficient results indicated that the samples extracted from SCG most likely had a lower molecular weight and degree of condensation as well as a lower lignin content. The calculated HB values suggested a lower hydrophobicity of the obtained humic-like substances (HLSs) compared to the reference samples, which may indicate their higher bioactivity and beneficial effects on the development of plant root systems.

The results obtained in this study suggest that both compost and spent coffee grounds can, to some extent, serve as renewable alternatives to raw materials that have traditionally been the main sources for humic preparations. Thus, their wider application in the production of natural agrochemicals could contribute, among other things, to the protection of peatlands by significantly reducing their industrial exploitation. A challenge in replacing lignite or peat with renewable raw materials in humic substance production technology may be the variability in their composition, which is determined by the conditions of their formation or origin. A potential solution in this regard could be further research on the impact of composting conditions and the raw materials used in this process on the quality and quantity of humic-like substances obtained in the produced compost.
